# Book Reviews

**Published:** 1855-09

**Authors:** 


### THE EPITOME OF SANITARY LITERATURE.

[UNDER the above title we shall from time to time submit for perusal brief notices of the works which come to hand for review. In these notes it will be our object to refer specially to the most practical and interesting portions of the works under consideration. The epitome thus constructed will be allowed to take the place of one or more critical reviews; but the mere reference to any work in this cursory way will not necessarily prevent the same work from being afterwards considered in a more general and more comprehensive article.]

### THE SMOKELESS FIRE-PLACE.*

THE smokeless fire-place is thus briefly described. A day's charge of coal is placed in a box fitted to, and beneath the grate, and is raised as wanted on a moveable false bottom or piston by the poker, used as a lever, acting on notches in the piston-rod made to admit its point, a racket-catch supporting it at the desired height on the withdrawal of the poker. The piston-rod fits accurately in an opening in the true bottom of the box, so that the air supplying combustion operates only on the coal exposed above the box and in the grate.

The modes of lighting, replenishing, ash-removal, and extinguishing are also given, and the fire so made is said to be very tenacious of life. Dr. Arnott's work also treats of the prevention of waste of fuel, and defective warming and ventilation; of ordinary dwelling ventilation; of ventilation on a large scale by pump, fan-wheels, shafts, &c.; of warming in general; of ventilation and warming by transfer of heat from used air to fresh air entering; and of a new self-regulating fire. The work throughout is written in Dr. Arnott's classical and forcible style; but we deeply regret to see in it (at page 42) an uncalled for and ungenerous attack on Dr. Chowne and his system of ventilation. In his severe strictures on Dr. Chowne's labours and knowledge, Dr. Arnott has entirely misstated the views and opinions held by that gentleman; and, having first made a statement to the effect that Dr. Chowne attributes the passage of air through tubes to their mere bent or syphon-shape, he has then torn the idea to pieces with a merciless hand. The truth however is, that Dr. Chowne has never based his principle of ventilation on the question of the shape of the tube, but simply on the curious fact which we have described in a succeeding page. The only mistake Dr. Chowne has made is in using the word "syphon" at all.

### OFFENSIVE TRADES AND SPREADING DISEASES.†

DR. SNOW strives in this pamphlet to support the idea that mere

* On the Smokeless Fire-place, Chimney-valves, and other means, old and new, of obtaining healthful warmth and ventilation. By NEIL ARNOTT, M.D., F.R.S.

† A Letter to the Right Hon. Sir Benjamin Hall. By JOHN SNOW, M.D.

bad smells are not in themselves capable of giving rise to specific diseases. He does not advocate the existence of offensive trades in populous districts, but suggests that all nuisances of this kind should be removed, on the mere ground that they are nuisances, *not* on the assumption that they give rise to special disorders. This is an extreme opinion, and contrasts remarkably with the prevalent feeling on this subject. Whether it be right or wrong, however, it is certainly not more extravagant, and is much more logical, than many of the statements and arguments of the ultra-anti-nuisance people, who can trace the origin of scarlet fever back to a handful of decomposing turnip-tops, or small-pox to a bone-shed. That nuisances are an abomination, and that they have an injurious effect on health is certain; for man is an oxygen breather only, and ought no more to breathe a nuisance than eat a nuisance. But it is the grossest of all quackery and assumption, to suppose, as many do, that the mere study of the nuisance question can settle the various points connected with the origin and course of specific diseases. So far, we agree with the author before us.

### THE FINANCIAL ASPECTS OF THE SANITARY QUESTION.*

NOTHING is now more obvious to the world at large than the fact that health is wealth—wealth in a variety of ways: in the reduction of taxation; in the saving of the doctor's bill; in the saving of happiness. Mr. Chadwick, in the excellent treatise in hand, has spoken on these facts, and has proved them. He takes, for the purpose of illustrating his point, a comparison between the townships of Salford and of Manchester, and those of a neighbouring township, Broughton. In Salford, thirty-one persons per thousand die annually; in Manchester, even a larger number; whilst in Broughton only fourteen persons in a thousand die yearly.

To account for this by reference to the situation or site of the buildings in each township would be useless; a great portion of the people in Broughton live at as low a level as in Salford, and some localities in Salford are as healthy as any in Broughton.

The cause of the difference lies in the circumstance that in Salford there are masses of over-crowded, ill ventilated, damp and undrained houses, that there is a want of water and other domestic conveniences, and a consequent absence of cleanliness, which leads to want of self-respect, to drunkenness, to disease, and crime.

There is also great over-crowding, except in the twenty-seven common lodging-houses now registered in Salford, and in the other public institutions. The over-crowding in other parts will exist, says Mr. Chadwick, until the working classes themselves understand this subject, and begin to ask and to require proper sanitary regulations, and refuse to occupy, at any price, badly drained, damp, ill-

* The Financial Aspects of the Sanitary Question. A Lecture by DAVID CHADWICK, Esq., C.E., Treasurer of the Borough of Salford.

ventilated, and over-crowded houses; as sure as the working classes demand this, so sure will a good supply of better houses, without any material advance in the rents, be provided. These are the true facts. For every death, there are twenty-five cases of illness on an average; each death costs on an average £60, including funeral expenses, medical attendance, loss of labour, and the like. Judging from analogy, there are in Salford 700 deaths annually from preventable causes alone; hence, to remove these causes might ensure an annual saving of a sum of money exceeding £40,000. To effect this saving, the main thing required is, that the working classes should understand what are the sanitary requirements to secure health and comfort in their homes.

### GYMNASTIC EXERCISES.*

According to the writer of these rules, the health of the present generation is on the wane. We are all going on to a premature decrepitude and death. Sad prophecies these, and still sadder, did not the prophet who brings them bring also the remedy. Dr. Lotsky's grand *modus curandi* is gymnastic exercise. He would erect "schools of strength and health" everywhere—in private gardens, in public gardens, in squares, in open places. Here might all congregate, and here might young and old, gentle and simple, revive the days of old Sparta, and practise in the wrestle and the race. The very simple parlance, says our enthusiastic author, "he is a strong lad", "she is a strong girl", points out the way which we have to take. The health of the world is not on the wane, but it admits of much improvement, and Dr. Lotsky's idea of gymnastics is excellent in that direction. If he could succeed in putting down dancing academies, and in pushing the world on some fifty years, he might stand a chance of having his idea realised, and of finding himself king of the gymnasia.

### MOVEMENT OF THE ATMOSPHERIC AIR IN TUBES.†

In a paper read before the Royal Society by Dr. Chowne, experiments are related to show the curious fact that the ordinary condition of atmospheric air within vertical tubes, open at both extremities, is one of continued upward movement. The results also show that the ordinary temperature of the air, within tubes vertically placed and open at both ends, is higher than that of the air external to them; and that the presence of aqueous vapour in the atmosphere is essential to the production of the current through the vertical tube. The experiments are interesting, useful, and philosophically contrived.

---

* Rationale and Rules of Muscular (Gymnastic) Exercise. By Dr. J. Lotsky. A Table for suspension in rooms.

† Experimental Researches on the Movement of the Atmospheric Air in Tubes. By W. D. Chowne, M.D. (Phil. Trans.)

### HARROGATE WATERS.*

THERE are four classes of what are called "medicinal waters" at Harrogate.

CLASS I. Strong sulphur waters, two of which are commonly resorted to: "the Old Well and the Montpellier Strong Sulphur Well." These waters are stimulant, aperient, and diuretic.

CLASS II. The mild sulphur waters, of which there are seventeen springs. These waters are diuretic, alterative, and diaphoretic.

CLASS III. Saline chalybeates; one spring of which is in the Montpellier pump-room, the other in the Cheltenham pump-room. They are tonic, aperient, diuretic, and deobstruent.

CLASS IV. Pure chalybeates, four springs. The waters are tonic and diuretic.

### THE VARYING FORMS OF THE HUMAN CRANIUM.†

THE curious and interesting question of the variations of size and shape in the human skull, and the effects of these modifications on the social condition of mankind, have been well and forcibly discussed by Mr. Dunn. He argues, that if we admit the influence of want, ignorance, and neglect, in accounting for the debasement of the savages of our own great cities; and if we witness the same effects occuring under the same conditions among the Bushmen of Southern Africa, we can scarcely hesitate in admitting that the long continued operation of the same agencies has had much to do with the psychical as well as the physical deterioration of the Negro, Australian, and other degraded savages. It will be only when the effect of education, intellectual, moral, and religious, shall have been fairly tested by the experience of many generations, in conjunction with the influence of a perfect equality in civilisation and social position, that we shall be entitled to speak of any essential and constant psychical difference between ourselves and the most degraded beings in a human form.

### THE CLIMATE OF MADEIRA.‡

THE value of change of climate for consumptive and other invalids involves many questions not yet settled, and which never will be by mere controversial letters. Certainly the opinion is now fast gaining ground amongst the medical profession, that change of climate, as a remedial measure, has been seriously overestimated; and that persons too often have been sent away *to die* far from their homes and friends. The effects of climate on disease would form a grand subject for governmental inquiry.

---

* Harrogate and its Resources. Chemical Analysis of its Medicinal Waters. By A. W. HOFFMANN, F.R.S.

† Observations on the varying Forms of the Human Cranium, in Relation to the Outward Circumstances, Social State, and Intellectual Condition of Man. By ROBERT DUNN, F.R.C.S.

‡ A Letter on the Climate of Madeira. By JAMES MACKENZIE BLOXAM, Esq. Second Letter.

### THE SOUTH STAFFORDSHIRE COLLIERY DISTRICT.*

Mr. Girdlestone observes that nowhere does the gross neglect of every sound sanitary principle tend to degrade the people physically and socially, morally and religiously, more than in South Staffordshire. He suggests for the remedying of this condition that sanitary reform should be promoted in the dwellings of the workmen, and exemplified in the 'masters' works and offices. Baths and washhouses would be doubly valuable where so much black dirt is incidental to the work; and cheaply attainable where coals cost least, and hot water is poured by tons into the canals, and steam blown into the air perpetually. Model lodging-houses would be especially desirable, owing to the resort of single men to the works, and the inconvenience resulting from lodging strangers in families without suitable apartments. In all such measures, besides the direct good they would effect, they would afford a proof of the masters caring for the men. On this ground foot-paths ought to be kept with greater care, and the works might often be better arranged for the convenience of the workers. They might be supplied with waiting-rooms, as already suggested, where temperate refreshments might in some cases also be provided, to which might be added a reading-room and library, and such unobjectionable sources of amusement as would compete with the skittle-ground, the fair, and the race-course.

These are Mr. Girdlestone's thoughts and suggestions. Happy, say we, is the neighbourhood possessing a pastor who preaches such practical, humanising, and benevolent discourses. There is more of the essence of true and earnest religion in such utterances, than in all the learned theological controversies that have rent the world of thought into a thousand divisions for ages past. When the clergy as a body shall follow in the track of our author, and begin systematically to teach physical improvements as well as moral, a true social reformation will not long wait to be revealed.

### THE WATER OF HOLYWELL AND TADMARTON HEATH.†

The question having been raised as to how Banbury should be best supplied with water, the water of Holywell, at Tadmarton Heath, a place five miles from Banbury, was submitted to analysis by Mr. Beesley, a most excellent chemist and a gentleman of great general and scientific acquirements. At the present time the town of Banbury is almost exclusively supplied with water from wells, into some of which the drainage of the sewers and other receptacles of refuse finds access, and from cisterns, in which rain-water collected from the roofs of houses is stored for use,—a supply which is always

---

* The South Staffordshire Colliery District: its Evils and their Cure. By the Rev. Charles Girdlestone, M.A.

† A Chemical Analysis of the Water of Holywell and Tadmarton Heath, with an Enquiry as to its Fitness for the Water Supply of Banbury. By Thos. Beesley, F.C.S.

contaminated with organic matter and soot. Under these circumstances, it is obvious that the town of Banbury ought to receive its water from some other source, and Mr. Beesley shows that the Holywell spring is well adapted for this purpose. It contains scarcely a trace of organic matter, and the degree of hardness is but 12·1°. The writer observes, that water for the supply of a town should possess an abundant and regular flow, a sufficient elevation of the source to allow of its complete distribution by its own pressure alone, a temperature unaffected by the changes of season, perfect limpidity, rendering unnecessary any artificial filtration, and the highest attainable degree of chemical purity and softness. This is really a perfect summary of all the requirements in regard to water.

### SANITARY STATE OF DUDLEY.*

In these reports Mr. Houghton has given an excellent epitome of the sanitary state of Dudley. His account shows, that in few towns in England is sanitary knowledge more required. It appears that pigs and pigsties are the great nuisances of Dudley, and we fear from the report that very little progress has been made in that town in sanitary education. We hope Mr. Houghton will be supported in his endeavours to impress on his townsmen the motto of our journal, that health is wealth, and happiness too. The mortality of Dudley is very high, the average giving to each person little more than half the duration of life in healthy towns. The number of deaths per thousand is not less than 36·03 per annum.

### THE SCOTTISH METEOROLOGICAL ASSOCIATION.†

Through the exertions of Sir John Stewart Forbes, of Port Sligo, the Scottish Meteorological Association has commenced with much promise of success. At the first meeting, the Marquis of Tweeddale being in the chair, various speakers referred to the importance of meteorology in regard to agriculture and to health. Professor Rogers, of Boston, U.S., made the following remarks. "It is quite notorious, in the United States, that a prolonged drought in August and September is the almost sure forerunner of a prevalence of autumnal epidemics, and the cause of this influence is supposed to be the great deterioration which the culinary waters undergo when the rains are long withheld, and the moisture within the soil acquires a concentrated excess of the soluble mineral elements of the earth and rocks. This effect is specially marked in those Southern and Western States where the strata are horizontal and unbroken, and where, therefore, the surface waters percolate to but a shallow depth." We do not before recollect having heard this theoretical explanation of the cause of autumnal epidemics, and we are unfortunately not told what are the epidemics thus produced.

---

* Three Reports on the Sanitary State of Dudley. By J. H. Houghton, Esq., Officer of Health.

† Report of the Preliminary Meeting of the Scottish Meteorological Association.

